# Substrate Recognition and Motion Mode Analyses of PFV Integrase in Complex with Viral DNA via Coarse-Grained Models

**DOI:** 10.1371/journal.pone.0054929

**Published:** 2013-01-24

**Authors:** Jianping Hu, Ming Liu, Dianyong Tang, Shan Chang

**Affiliations:** 1 Department of Chemistry and Life Science, Leshan Normal University, Leshan, China; 2 Beijing Institute of Biotechnology, Beijing, China; 3 College of Informatics, South China Agricultural University, Guangzhou, China; Weizmann Institute of Science, Israel

## Abstract

HIV-1 integrase (IN) is an important target in the development of drugs against the AIDS virus. Drug design based on the structure of IN was markedly hampered due to the lack of three-dimensional structure information of HIV-1 IN-viral DNA complex. The prototype foamy virus (PFV) IN has a highly functional and structural homology with HIV-1 IN. Recently, the X-ray crystal complex structure of PFV IN with its cognate viral DNA has been obtained. In this study, both Gaussian network model (GNM) and anisotropy network model (ANM) have been applied to comparatively investigate the motion modes of PFV DNA-free and DNA-bound IN. The results show that the motion mode of PFV IN has only a slight change after binding with DNA. The motion of this enzyme is in favor of association with DNA, and the binding ability is determined by its intrinsic structural topology. Molecular docking experiments were performed to gain the binding modes of a series of diketo acid (DKA) inhibitors with PFV IN obtained from ANM, from which the dependability of PFV IN-DNA used in the drug screen for strand transfer (ST) inhibitors was confirmed. It is also found that the functional groups of keto-enol, bis-diketo, tetrazole and azido play a key role in aiding the recognition of viral DNA, and thus finally increase the inhibition capability for the corresponding DKA inhibitor. Our study provides some theoretical information and helps to design anti-AIDS drug based on the structure of IN.

## Introduction

Integrase (IN) is one of the three key enzymes involved in the life cycle of the HIV-1 virus. The full-length HIV-1 IN comprises 288 residues, which can be divided into three domains, i.e. the N-terminal domain (NTD, residues 1∼50), the catalytic core domain (CCD, residues 51∼211) and the C-terminal domain (CTD, residues 212∼288). NTD contains a conserved “HHCC” motif binding with a Zn^2+^ ion and can promote enzymatic multimerization [1], [2]. CCD is composed of six α-helixes and five β-sheets, and also contains an absolutely conserved D-D-35-E motif (i.e. Asp64, Asp116, Glu152) chelated by two Mg^2+^ ions. CCD mainly serves as endonuclease and polynucleotideyl transferase [3]–[5]. CTD has relative poor conservations and is found to strongly and non-specifically bind with different DNA sequences [6], [7]. Many in vitro experiments, such as site-directed mutagenesis has revealed that sole CCD is sufficient for the disintegration reaction [8], [9]. IN catalyzes the integration reaction in two steps. The first step is termed as 3′ end processing [10], in which two nucleotides are removed from 3′-end of each strand of viral DNA to produce a functional base end (i.e. Cytosine-adenine, CA). The second step named as DNA strand transfer (ST) occurs in the nucleus [11], where the CA end of viral DNA is covalently joined to the host DNA.

IN strand transfer inhibitors (INSTIs) are the major anti-HIV-1 IN lead compounds. Diketo acid (DKA) molecule is one of the important INSTIs [12]–[15]. These molecules have been shown to selectively inhibit the IN ST step by chelating divalent Mg^2+^ ions [16]. By now, only one active drug against HIV-1 IN, raltegravir (RLV), is approved by FDA. RLV belongs to the DKA family of INSTIs [17]. Unfortunately, the lack of detailed structural information for the interactions between IN and its cognate viral DNA largely hampers anti-AIDS drug design based on the structure of HIV-1 IN.

Recently, Hare et al reported the crystal structure of the full-length IN from the prototype foamy virus (PFV) in complex with its cognate viral DNA and RLV inhibitor. PFV IN has highly structural and functional homology with HIV-1 IN [18]. BLAST program was used to calculate homologous degree between 151 amino acids of HIV-1 IN CCD and 145 amino acids of PFV IN CCD. The region possessing the maximum homology for PFV IN is from Lys180 to Leu234, which just includes catalytic loop (Glu207∼Glu221) and DNA-binding site (i.e. Asp185 and Glu221, etc.) [19]. Availability of this crystal structure enabled us to explain the mechanism of strand-transfer inhibitor action. Some previous experimental and molecular modeling studies [10], [20] showed that IN undergoes significantly conformational and motion mode change when it plays biological function or binds with INSTIs. In addition, Valkov’s biological experiment [21] shows that the in vitro ST activity of PFV IN is ablated in the presence of >200 nM of RLV or other HIV-1 INSTIs. In other word, PFV IN is sensitive to HIV-1 IN inhibitor including DKA molecules.

However, what are the certain conformational change and the correlation with the biological function? How are PFV IN-DNA systems recognized by HIV-1 INSTIs? All of these important questions still remain elusive. Based on the PFV IN-DNA crystal structure, the aim of this article is to investigate the conformational change of PFV IN and interpret the interactions between PFV IN and INSTIs via coarse-grained model including Gaussian network model (GNM), anisotropic network model (ANM) and molecular docking methods. Finally, the correlation between experimental IC_50_ value and the calculated docking energy is briefly analyzed. This work provides some theoretical information for anti-AIDS drug design based on the structure of PFV IN-DNA complex.

## Results and Discussion

### The Structure of PFV IN-DNA System

**Figure 1:**
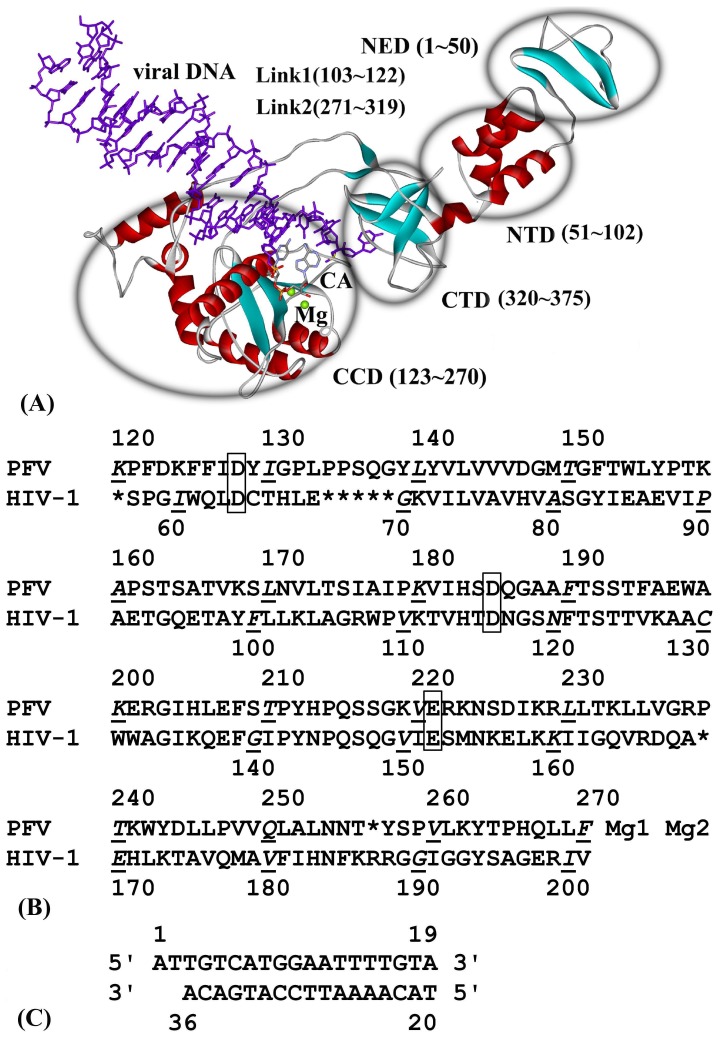
Structure and sequence alignment of IN and viral DNA (PDB code: 3L2T). (A) The structure of PFV IN and viral DNA. (B) The sequence alignment of the CCD for PFV and HIV-1 IN. (C) The sequence of viral DNA.

Figure 1 shows the spatial structure of PFV IN-DNA (PDB code: 3L2T), the sequence alignment of CCD between PFV and HIV-1 IN, as well as the sequence of viral DNA. As shown from figure 1A, the structure of PFV IN is composed with NTD, NTD extension domain (NED), CCD, CTD, L1 (the link region between NTD and CCD) and L2 (the link region between CCD and CTD).

### The Reliability of Coarse-grained Models

**Figure 2:**
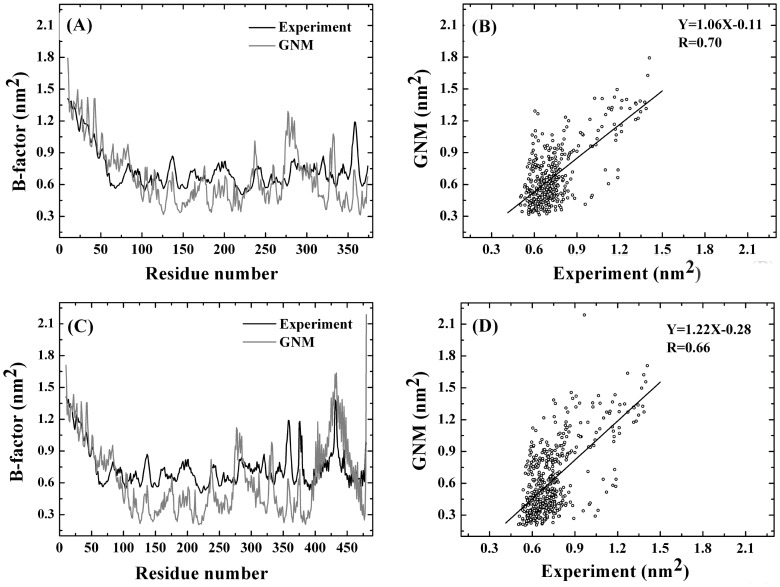
Comparison and correlation of the experimental and calculated B-factors for the PFV IN and IN-DNA systems. (A) Experimental and calculated B-factors for PFV IN system. (B) Correlation of experimental B-factors and the calculated values for PFV IN system. (C) Experimental and calculated B-factors for PFV IN-DNA system. (B) Correlation of experimental B-factors and the calculated values for PFV IN-DNA system. The number of 10∼374 represents the id of C_а_ atoms for PFV IN and the number of 375∼480 denotes viral DNA. Every base is described by P, C4’ and C2 atoms. Because the 5′ end of viral DNA (i.e. A1 and T20) has no P atoms, 106 (36×3−2 = 106) is enough for defining viral DNA.

B-factor can be used to represent the flexibility of protein. Figure 2 shows the calculated B-factor for PFV IN and IN-DNA systems, as well as the comparative analysis with experimental data. As shown from figure 2, the correlation coefficient between the calculated and the experimental B-factors of the PFV IN and IN-DNA systems are 0.70 and 0.66, respectively. High correlation coefficients indicate that the results gained from GNM and the following ANM analysis should be reliability. The calculated B-factor through GNM is slightly higher than those corresponding experimental data, which may result from the crystal stacking effect restricting atoms to some extent. In addition, the correlation coefficient is 0.57 when only taking viral DNA into account, which may because that viral DNA possesses high flexibility by itself after obvious structure deletion process.

### Fast and Slow Motion Modes

The motions of bio-macromolecule can be divided into fast and slow modes. The fast motion modes have local harmonic motion character corresponding to narrowly geometric irregularity. Thus, residues acting in the fast modes are thought to be kinetically hot residues and important for molecular recognition [22]–[24]. The slow motion modes have a long-wavelength collective character, which represents functionally un-harmonic motions [24]. Especially, the first slow motion mode is important for bio-macromolecule to exert its biological function.

**Figure 3:**
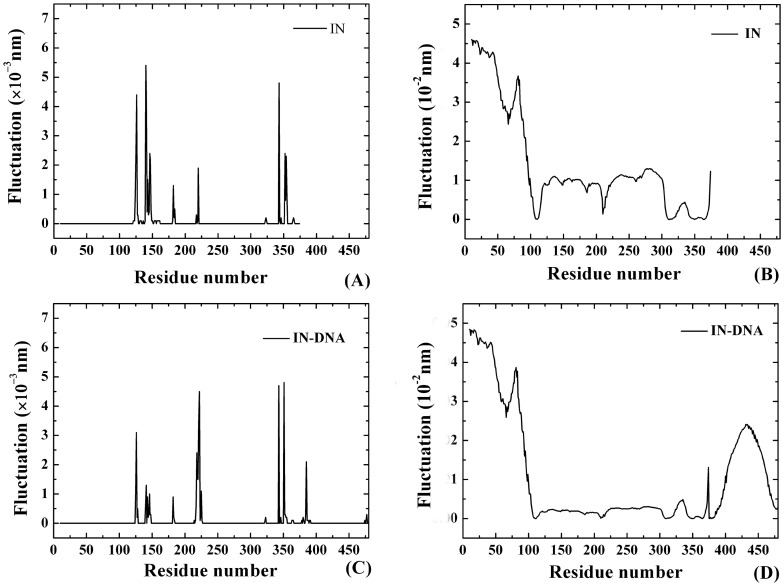
The fast and slow motion modes of PFV IN and IN-DNA systems. (A) Fast motion mode of IN. (B) The first slow motion mode of IN. (C) Fast motion mode of IN-DNA. (D) The first slow motion mode of IN-DNA. The definition of residue number is the same with that of Figure 2.

On the basis of the PFV IN-DNA crystal structure, the contact residues of functional CA end (i.e. C35-A36 in this work) were given by an in-house VMD Tcl/Python script. Here, the residues of the protein in the neighborhood of 0.4 nm of CA bases are defined as contact residues, which is important for the specific recognition of viral DNA [25], [26]. The results show that the contact residues are mainly distributed from Glu221 to Lys228. Figure 3A shows the fast motion distribution of PFV IN system via GNM method. The peak value of fast motion exists in the following amino acids, i.e. Phe126, Leu140, Ile182, Val220, Val343 and Val352. All of those residues lie in the inner part of CCD and CTD having the most tight atom-atom interactions. After association with viral DNA, there is an obvious change of fast motion at Arg222. In detail, the peak values of Arg222 and its neighbor residues significantly rise (see figure 3C). The result shows that this region participates in recognition of DNA. Obviously, the modeling results agree well with the experimental data. In other words, the information of binding site is fully and correctly represented via the GNM methods, which reconfirms that the coarse models of GNM and ANM are reliably used in this work.

Figure 3B and D show the distribution of the first slow motion for the PFV IN and IN-DNA systems, respectively. As shown from figure 3B and D, both NED (residues 1∼50) and NTD (residues 51∼102) of the two systems exhibit significantly slow motion. The CCD (residues 123∼270) participating in the recognition with DNA possesses obviously slow motion in the IN systems, while the scope of slow motion for this region significantly decreases after the association with viral DNA. In addition, the motion direction and motion-function correlation of these slow motion modes will be discussed later.

### Motion Correlation Analyses

**Figure 4:**
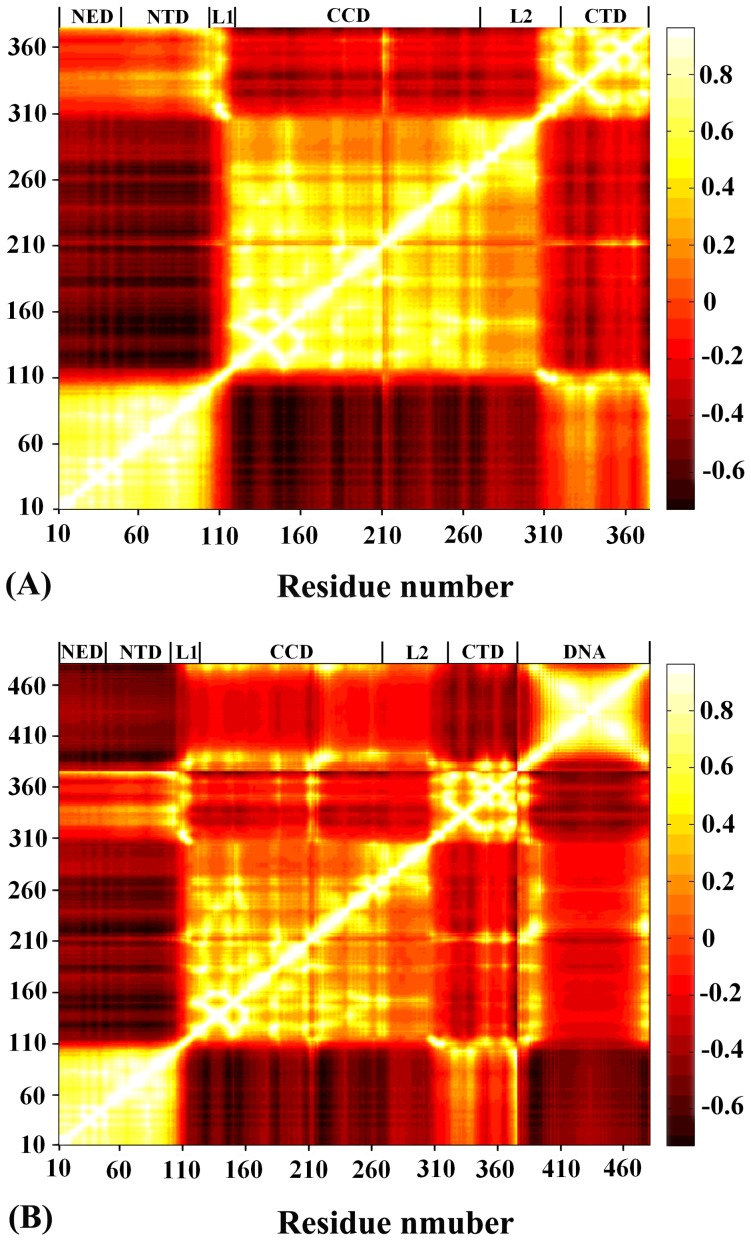
Cross-correlation map of PFV IN (A) and IN-DNA. Cross-correlation map calculated using ANM for PFV IN and IN-DNA systems are shown in panel (A) and (B), respectively. The definition of residue number is the same with that of Figure 2.

Figure 4 shows the motion correlation of each domain (i.e. NED, NTD, CCD and CTD) for the PFV IN and IN-DNA systems. As shown from figure 4, the association with viral DNA only slightly influence the motion correlation of IN. In detail, IN has three distinct motion correlation regions (i.e. NED/NTD, CCD and CTD), which possess obvious inner motion positive correlation. There is obvious negative motion correlation between CCD and NED/NTD, while the link parts (i.e. L1 and L2) only have relatively weak motion correlation. In addition, the bases having highly positive motion correlation with CCD mainly lie in G4 and C35-A36, as well as their neighbor region. There is highly positive motion correlation for viral DNA, which may be resulted from the obvious revolving motion of this molecule. The similar phenomena are reported in Hu’s previous study [20], where both free viral DNA and the DNA in complex with HIV-1 IN dimmer have an obvious revolving motion around its axis. It is speculated that the revolving motion may be a universal motion for a DNA segment in water.

To sum up, both DNA-free and DNA-bound IN have three distinct motion domains, which have highly inner positive motion correlation. The functional C35-A36 end bases have obviously positive motion correlation with the CCD of IN, which shows that a completely functional unit is composed of the CA end and CCD.

### The Direction of Slow Motion

**Figure 5:**
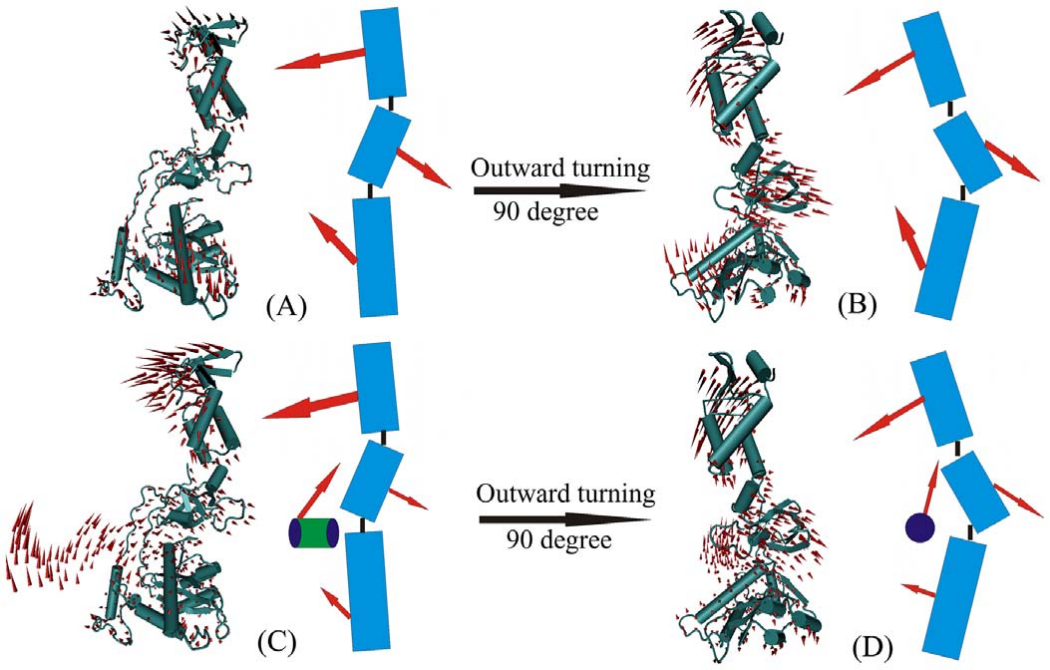
The first slow motion modes of PFV IN and IN-DNA. The front and side face views for the first slow motion modes of PFV IN and IN-DNA systems are illustrated in panel (A, C) and (B, D), respectively. The slow motion modes are shown with cone model. The length of the cone is correlative with the motion magnitude and the motion direction is depicted with the orientation of the cone. The NED/NTD, CTD and CCD are denoted by three red rectangles in the sketch map.

Motion correlation analysis only provides the relationship of moving direction for each domain of PFV IN. We are also interesting in the absolute moving directions of PFV IN and IN-DNA systems, which is an important question for investigating the biological function of the enzyme. Figure 5 shows the moving direction of the first slow motion for the PFV IN and IN-DNA system. As shown from figure 5, NTD/NED has the largest moving scope compared with the other domains for the two systems, which is consistent with the information gained from figure 3B and D. The moving direction of NED/NTD is pointing to the DNA binding site. This motion mode may aid the integration process of IN to some extent, decrease the contact area with water, and finally increase the inner stability of this enzyme. The moving direction of CCD is also pointing to DNA binding site in the PFV IN system, and may tend to bind with DNA. However, the association with DNA largely weakens this motion, which is replaced by that viral DNA has an obvious motion tendency toward the center of the PFV IN-DNA system. In fact, the CCD of PFV IN-DNA system has a weaker scope of motion compared with the free IN, which are mentioned in figure 3B and D. The moving direction of CTD is opposite to that of CCD and NED/NTD and points to molecular outside. The opposite motion direction are also clearly seen from the above figure 4. The motion of CTD accompanies CCD/NTD/NED to bend a bit and bind with viral DNA.

In a word, the first slow motion of PFV IN slightly favors binding with viral DNA. Both IN and IN-DNA systems have similar motion modes. The slight difference is that the motion of the CCD for the latter system is weaker than that of PFV IN system. It is proposed that the binding ability with viral DNA is the intrinsic property of PFV IN, which is determined by its specific structural topology.

### Molecular Docking Experiments

**Figure 6:**
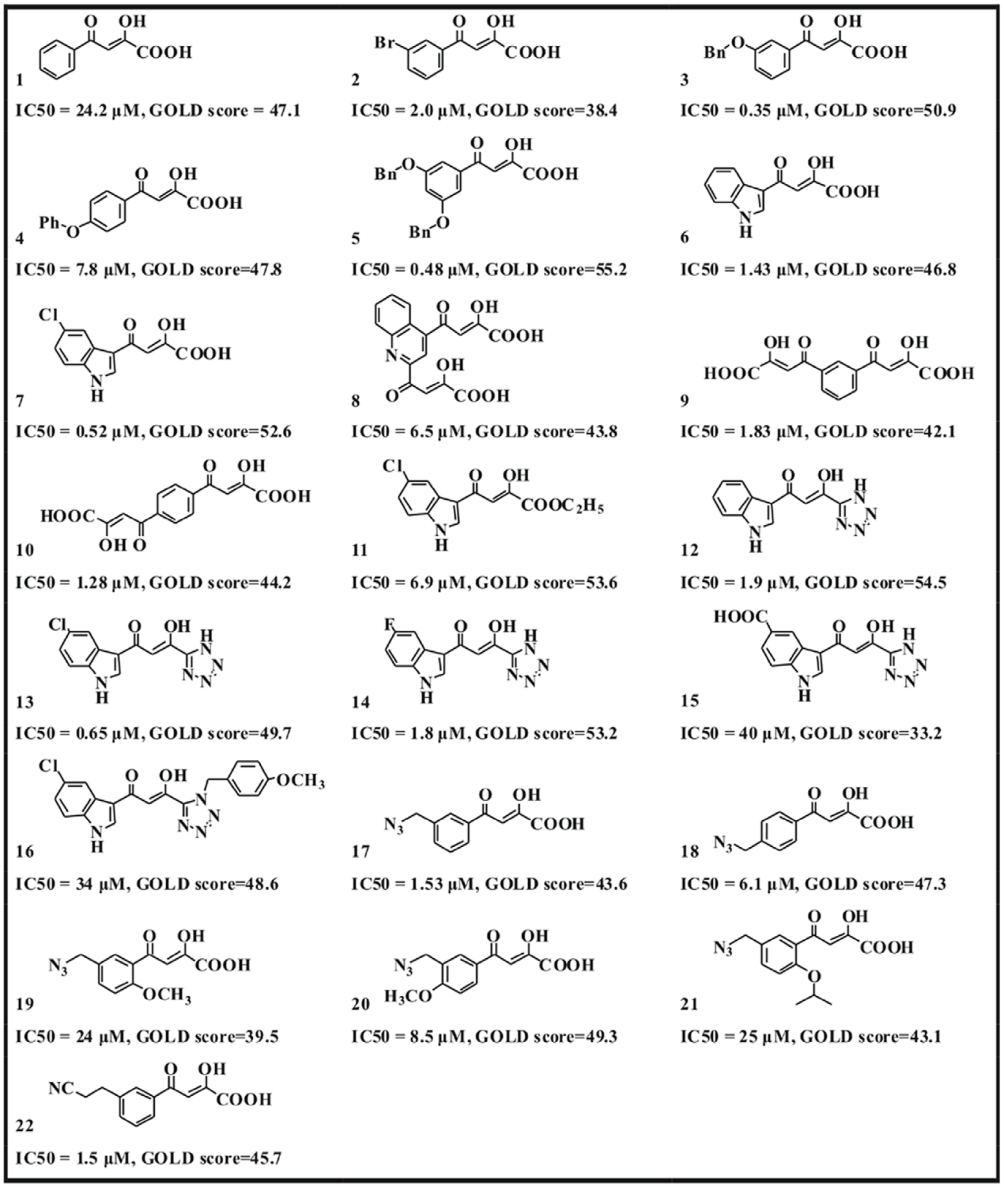
Structures and measured activities of a series of DKA inhibitors used for the docking study.

There is a lack of the full-length HIV-1 IN-viral DNA crystal structure, and PFV IN has the highly functional and structural homology with HIV-1 IN. We used PFV IN as a surrogate model for HIV-1 IN to perform the molecular docking experiments. Here, the receptor of molecular docking is PFV IN-DNA complex structure obtained from ANM, and the ligands are a series of DKA inhibitors. It is well known that reported that the possible inhibition mechanism of DKA inhibitors is to compete with the association of viral DNA with IN, thus it is better to perform molecular docking experiment between DNA-unbound IN and inhibitors [27]. Recently, Hare group [18] has reported the crystal structure of PFV IN complexed with its cognate viral DNA and RLV inhibitor. It proves that inhibitors and viral DNA can coexist to bind with IN system. We believe that the docking between inhibitor and DNA-unbound IN reflects inhibitor inhibiting 3′ end processing step (i.e. inhibiting IN to binding with viral DNA). The docking between inhibitor and IN-DNA system imitates inhibitor inhibiting strand transfer step (i.e. inhibiting IN-DNA to binding with host DNA). All of the 22 inhibitors listed in figure 6 (the same as RLV inhibitor) are selective strand transfer inhibitors. Thus, we performed a series of molecular docking experiment between inhibitors and IN-DNA.

The possible binding modes of PFV IN-DNA with a series of DKA inhibitors were gained, and the feasibility for PFV IN-DNA used in anti-AIDS drug design is confirmed. It is worth mentioning that the starting acceptor conformation for molecular docking is obtained from ANM. The flexibility of biomacromolecule is partly taken into account, so the docking result may be more reliable. Figure 6 shows the structures and measured activities of 22 DKA inhibitors used for the molecular docking studies [28], [29]. As shown from figure 6, DKA inhibitors are composed of four subclasses, i.e. aryl-diketo acid (compounds 1∼7), bis-diketo acid (compounds 8∼10), diketo carboxylic ester (compounds 11∼16) and azido-diketo acid (compounds 17∼22).

**Figure 7:**
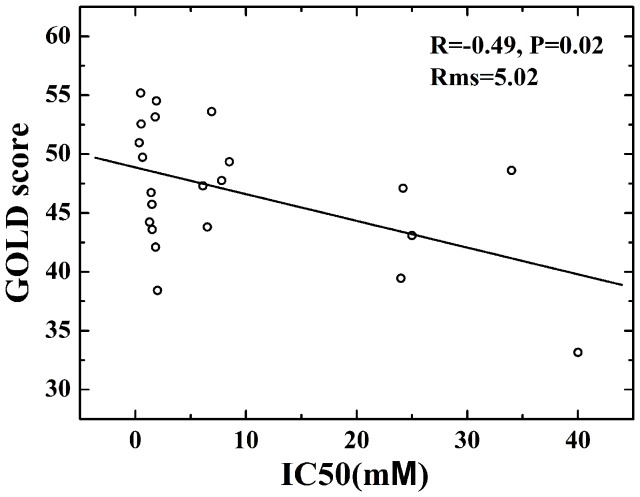
Correlation between the experimental IC_50_ values and the calculated GOLD score for a series of DKAs.

In order to assess the correlation between docking fitness function and biological activity, Figure 7 shows the plot of GOLD scores against measured IC_50_ values. As shown from figure 7, the regression analysis R, P and Rms values are −0.49, 0.02 and 5.02, respectively. It is found that there is a weak statistical correlation for 22 DKA samplings. Considering 10 µM to be a cutoff for activity, there are 17 actives and 5 in-actives (see figure 6). Here, we use the GOLD score of 40 or above as a predictor of activity. Total 16 compounds are selected from the 17 actives, while only 2 compounds are picked out among 5 in-actives. As for the drug screen of DKA inhibitors, it is proposed that GOLD score is a choosable indicator of activity, while the score function is not a suited parameter in distinguishing in-actives.

Huang et al perform some molecular docking between a series of DKA inhibitors and HIV-1 IN. It is found that the contact residues are composed of Asp64∼Thr66, Asp116, Ile141∼Gln146, Glu152, Asn155, Lys159 and Mg^2+^ ion [27], [30], [31]. Our molecular docking results show that the contact residues and bases of the 22 inhibitors are similar, which are composed of the following three regions: (1) D-D-35-E motif (i.e. Asp128, Asp185 and Glu221) and its near residues, (2) Two Mg^2+^ ions and the functional C35-A36 end of viral DNA, (3) catalytic loop region including Tyr212 and Pro214. All of the above three regions similarly exist in HIV-1 IN by referring to figure 1. Considering the similar binding modes of DKA or derivative inhibitors with HIV-1 IN and PFV IN, it is proved that the structure of PFV IN complexed with viral DNA can be effectively used in the anti-AIDS drug screen. DKAs in our work have four subclass structures and exhibit obviously different steric conformation. The four functional groups are the keto-enol moiety in the aryl-diketo acid inhibitors (compound 1∼7), bis-diketo acid (compounds 8∼10), the tetrazole group in diketo carboxylic ester (compounds 11∼16) and the azido group in the azido-diketo acid (compounds 17∼22) respectively.

**Figure 8:**
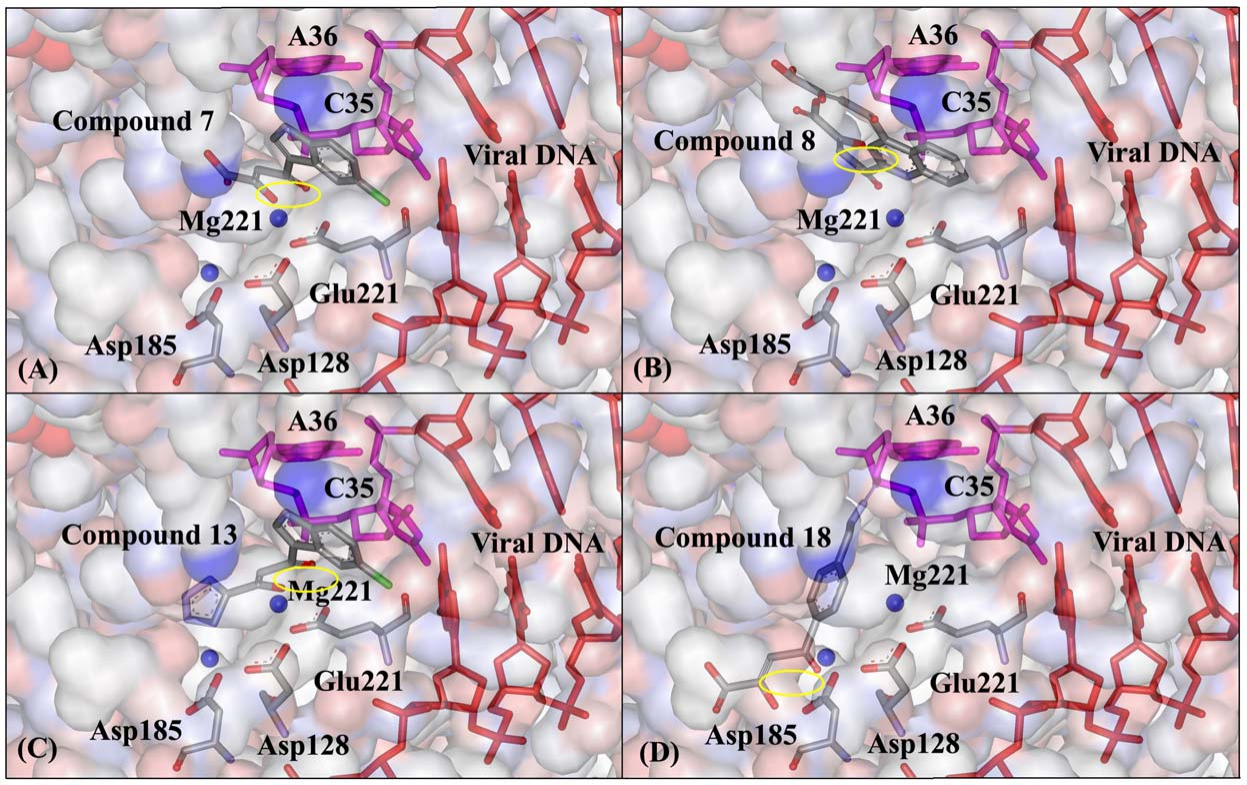
The representative binding modes of PFV IN-DNA with four inhibitors. (A) Compound 7. (B) Compound 8. (C) Compound 13. (D) Compound 18. The same orientation is adopted for PFV IN-DNA structure.

All the docking complexes of aryl-diketo acid inhibitors (compound **1∼7**) are superimposed, and the correlation between docked GOLD scores and experimental IC_50_ values is also fully considered. Finally, the binding mode between inhibitor 7 and PFV IN-DNA is determined as the most representative docking result (see figure 8A). As shown from figure 8 A, the keto-enol functional group is close to the Mg^2+^ ion (naming Mg221) near Glu221, and the aryl-ring is located near the active C35-A36 bases. It is worthy of noting that this docked result is different from that obtained through Autodock package, where the aryl-ring approaches Asp185 instead of C35-A36 bases [19]. The difference of docking results may root from different docking method and scoring function adopted. It is proposed that both the two binding modes objectively exist. After superimposing the three bis-diketo acid inhibitors (compounds 8∼10), the orientation of molecule 8 was selected as the most representative structure (see figure 8B). As shown from figure 8B, the orientation of one diketo-acid functional group is similar with that of compound 7, where the keto-enol group is well chelated by Mg221. The other diketo-acid functional group has obvious interactions with the active A36 base, and finally favors the tighter association between electron-withdrawing aryl-ring group and viral DNA. Figure 8C shows the binding mode between the representative diketo carboxylic ester containing tetrazole group (compounds 13) and IN-DNA. The electron-withdrawing tetrazole group is also close to Mg221. It is speculated that the biological function of tetrazole group is slightly favoring the ary-ring group containing Cl^-^ ion to approach viral DNA, which is similar with that of carboxylic acid group in compound 7. Figure 8D shows the binding mode of compound 18 (azido-diketo acid). As shown from figure 8D, the diketo group participates chelating Mg221, and the azido group deeply inserts into the double-strand viral DNA. In addition, there is a parallel alignment between the azido group and the aryl-ring of C35 base, which aids the formation of conjugated effect and increases the binding ability of azido-diketo acid inhibitors with viral DNA.

In a word, the functional groups of keto-enol, bis-diketo acid, tetrazole and azido in different DKA inhibitors play a key role in aiding the recognition by viral DNA and increasing the binding ability, which is important for these inhibitors (compounds 1∼22) to exert those biological functions. The binding mode gained only through molecular docking method is relative time-saving. In order to increase the structural precision of the docked system, quantum mechanics optimization and molecular dynamics simulation will be performed in other work.

## Methods

### Gaussian Network Model

In Gaussian network model (GNM), the three-dimensional structure of biomacromolecule is described as an elastic network. In the protein structures, C_α_ atoms are the nodes. In RNA/DNA structure, three-nodes model is adopted, and the three atoms (i.e. P, sugar C4’ and base C2 atoms) are nodes in a nucleotide. These nodes are connected by harmonic springs within a certain cutoff distance [32], [33]. In the GNM, the force constant is identical for all springs. Taking all contacting nodes into account, the topology of the network can be written as a 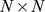 Kirchhoff matrix 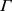. The off-diagonal elements are −1 if the nodes are within a cutoff distance, 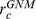 (7.3 Å is adopted in this work), and zero otherwise. The diagonal elements represent the coordination number of each node. The cross-correlation fluctuations between the *i*th and *j*th nodes are given by
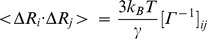
(1)where 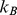 is Boltzmann constant, *T* is absolute temperature. The B-factor value, which is related to the mean-square fluctuation, can be calculated with 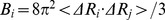. In the GNM, the cross-correlation is normalized as 
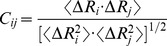
(2)


### Anisotropic Network Model

The GNM model can provide the amplitudes of node fluctuations but no information about the directions of the fluctuations [34], [35]. Then, the anisotropic network model (ANM) model is introduced, by which the orientation information of fluctuations is elicited. In the ANM, the motion mode of a structure is determined by a Hessian matrix *H*.
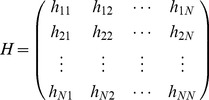
(3)


The element of *H* is composed of submatrix with size 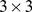. The submatrix 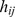 is
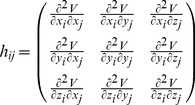
(4)


In the ANM, the cutoff distance 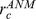 is set as 13 Å.

### Molecular Docking

A series of DKA inhibitors were docked to the binding site of PFV IN-DNA by means of CCDC’s GOLD (Genetic Optimization for Ligand Docking) software version 3.1.1 [36]. The binding region for the docking study was defined as an 8 nm radius sphere centered on the centroid of the two metal ions. 100 genetic algorithm (GA) runs were performed for each compound, and 10 ligand bumps were allowed in an attempt to account for mutual ligand/target fit. For each of the GA runs, a maximum number of 10^5^ operations were performed on a population of 100 individuals with a selection pressure of 1.1 atm. The number of island was set to 5, with a niche size of 2. The weights of crossover, mutation, and migration were set as 95, 95 and 10, respectively. The scoring function (i.e. GOLD score) implemented in GOLD was used to rank the docking positions of compounds, which were clustered together differing by more than 0.2 nm of root mean standard deviation (RMSD).

### Conclusions

The motion modes of PFV IN and IN-DNA systems are investigated with GNM and ANM methods. The B-factors calculated via GNM agree well with the experimental data, and the binding site of PFV IN with DNA is also correctly found, which confirm the reliability of coarse-grained models. The association of viral DNA only slightly influences motion correlation and direction for IN enzyme, and the sole difference is that the motion scope of the CCD for PFV IN-DNA system is weaker than that of IN. It is proposed that the binding ability with viral DNA is the inner property of PFV IN, which is determined by its intrinsic structural topology. In addition, a series of molecular docking experiments were performed between 22 DKA inhibitors and PFV IN-DNA obtained from ANM using GOLD package. The resulted scores have a highly correlation with experimental IC_50_ values. The binding modes of DKA inhibitors with PFV IN are similar with those with HIV-1 IN, which proves that PFV IN-DNA is a credible platform for HIV-1 IN inhibitor visual screen. This study may provide some help for anti-AIDS drug design based on the structure of IN.
